# Supplementary material for: Unilateral Hypofunction of the Masseter Leads to Molecular and 3D Morphometric Signs of Atrophy in Ipsilateral Agonist Masticatory Muscles in Adult Mice
Source: Int J Mol Sci. 2023 Sep 29;24(19):14740. doi: 10.3390/ijms241914740 (PMC10572689; doi:10.3390/ijms241914740)
Supplement: Supplementary file 1 [file ijms-24-14740-s001.zip › Supplementary_Material_Balanta-Melo_et_al_IJMS_2023.pdf]

Supplementary Material

Unilateral Hypofunction of the Masseter Leads to Molecular and 3D-Morphometric Signs of Atrophy in Ipsilateral Agonist Masticatory Muscles in Adult Mice

Julián Balanta-Melo, Andrea Eyquem-Reyes, Noelia Blanco, Walter Vásquez, Kornelius Kupczik, Viviana Toro-Ibacache, Sonja Buvinic.

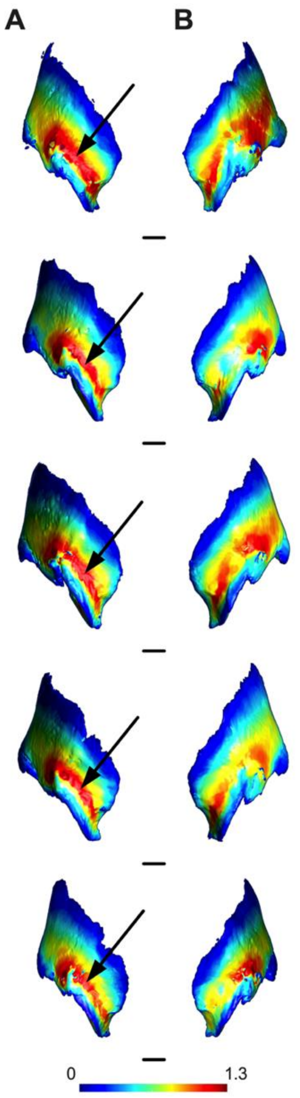

Figure S1. Comparative 3D depiction of *temporalis* thickness. A. experimental and **B.** control sides shows a local thickness increase of the muscles from **A.**, a consistent phenotype among all animals (black arrows). *Color-coded bar in mm*; *scale bar: 1 mm*.

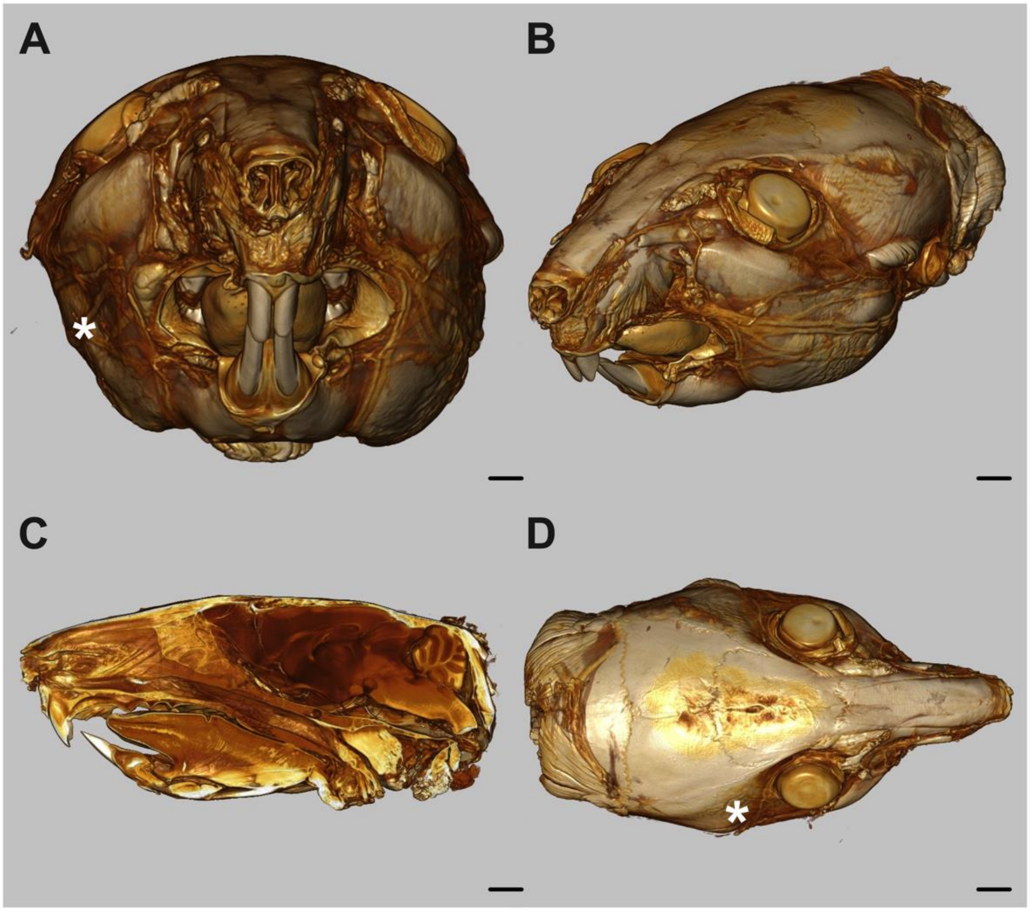

Figure S2. 3D rendering of large-volumetric µCT of a representative sample before processing. A. Frontal and **B.** rotated (after pitch, roll, and yaw) views, highlighting control side (*white asterisks*) for phenotypic comparison; **C.** Sagittal view after digital medial sectioning, showing efficient soft tissue staining even in internal structures such as the brain; **D.** Cranial view showing flattened phenotype in the right *temporalis,* in comparison to the control side. White asterisks: experimental side; scale bar: 1 mm.

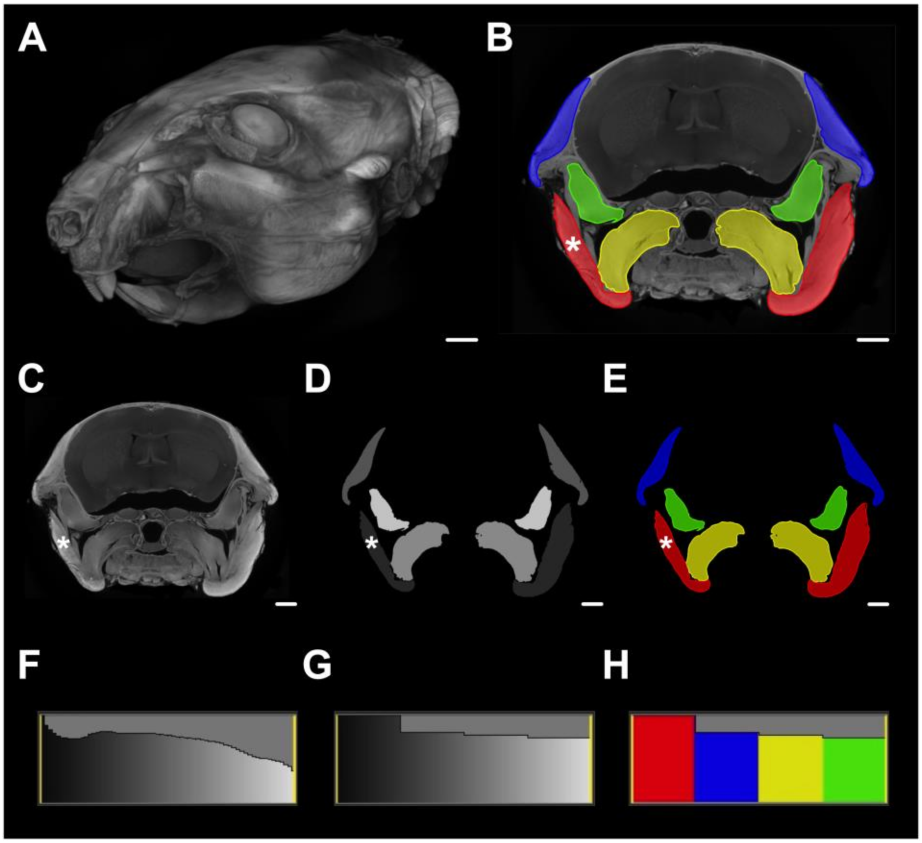

Figure S3. 3D depiction of downsampled µCT data and masticatory muscles segmentation. A. Rotated (after pitch, roll and yaw) view of full head from downsampled µCT dataset; **B.** Representative coronal section showing the contrast-enhanced microCT data as background with registered segmented regions of interest (Biomedisa output): Blue, *Temporalis*; Red, *Masseter*; Yellow, *Medial pterygoid*; Green; *Lateral pterygoid*; **C.** RAW slice, **D.** Output segmented data from Biomedisa visualized in DRAGONFLY 2021.3 and **E.** color-coded ROIs, at the same level (slice) of **C.** In **F-H.**, corresponding histograms from **C-E.**, respectively, showing spacial resolution differences, and highlighting how Biomedisa output from **D.** can be further improved by color coding (showing in **E.** and its corresponding histogram with 4 defined elements in **H.**). *Grey scale in F, from 0 to 255; Grey scale in G, from 0 to 5 (4 elements); Color scale in H, from 0 to 5 (4 elements);* White asterisks: experimental side; scale bar: 1 mm.
